# Patterns of Hepatocellular Carcinoma After Direct Antiviral Agents and Pegylated-Interferon Therapy

**DOI:** 10.7759/cureus.11565

**Published:** 2020-11-19

**Authors:** Tehreem Fatima, Hassan Mumtaz, Muhammad Hassaan Khan, Saad Rasool, Muhammad Tayyeb, Mobeen Z Haider, Syed T Hussain, Aamir Shahzad, Sundas Ali, Tanveer Hussain

**Affiliations:** 1 Internal Medicine, California Institute of Behavioral Neurosciences and Psychology (CIBNP), Fairfield, USA; 2 Urology, Guys & St. Thomas Hospital, London, GBR; 3 General Medicine, Surrey Docks Health Center, London, GBR; 4 Surgery, Kahutta Research Laboratory (KRL) Hospital, Islamabad, PAK; 5 Internal Medicine, Holy Family Hospital, Rawalpindi, PAK; 6 Internal Medicine, Mayo Hospital, Lahore, PAK; 7 Internal Medicine, King Edward Medical University, Lahore, PAK; 8 Medicine, Hamad General Hospital, Doha, QAT; 9 Internal Medicine, Holy Family Hospital/Rawalpindi Medical University, Rawalpindi, PAK; 10 Gastroenterology and Hepatology, Holy Family Hospital, Rawalpindi, PAK

**Keywords:** daa, direct anti-viral agents, direct anti-viral agents, peg-interferon, peg-interferon, hcc, hcc, hepatocellular carcinome, hepatocellular carcinome

## Abstract

Introduction: The impact of direct-acting antiviral agents (DAAs) on the development of hepatocellular carcinoma (HCC) is controversial and a part of the scientific community believes it as a biased interpretation of data. Many studies have reported an aggressive pattern of HCC after DAA use. In this study, we attempted to assess the changes in the pattern of HCC after treatment with DAAs or PI (PEG, pegylated-interferon).

Methods: A total of 37 HCC patients after DAA treatment and 21 HCC patients after PI treatment were included. The diagnosis of HCC was made and information about demographics, HCC infiltrative pattern, portal vein thrombosis (PVT), time at initial presentation, Child-Turcotte-Pugh (CTP) score, and Barcelona Clinic Liver Cancer (BCLC) stage were compared in the two groups.

Results: The total number of male patients in the DAA group was 62% while either gender was almost equal in PI. The age group of 40-60 was more prevalent in the DAA group while the PI group comprised more patients who were above 60 years. Patients in the DAA group presented after 3.35 years on average while patients in the PI group presented after about seven years. Most of the patients presented with the CTP stage of A. That is true for both groups. For BCLC staging, most of the patients had stage C, which means multiple lesions. At the initial presentation, most of the patients presented with multifocal lesions.

Conclusion: Our study found no significant difference in the initial presentation between both groups. However, HCC patients with prior DAA therapy presented early than those with PI therapy.

## Introduction

Hepatitis C Virus (HCV) which is a leading cause of chronic liver disease (CLD) and decompensated liver disease (DCLD) affects about 170-200 million individuals worldwide [1]. In Pakistan, approximately 10 million individuals are infected with HCV and this burden is expected to grow in the coming years [2]. Several efforts are being done by the officials in Pakistan to control the spread of disease as well as to timely manage all those suffering from it. A few years back, PEG (pegylated)-interferon (PI) was the only effective option for treating HCV infection. However, its outcomes were not satisfactory, resulting in high morbidity and mortality [3]. A couple of years ago, direct-acting antiviral agent (DAA) drugs have been approved by the Food and Drug Administration (FDA). These have transformed the treatment of HCV. DAAs have been so successful that sustained viral response (SVR) is achieved in more than 90% of the cases [1]. However, few studies have linked it to hepatocellular carcinoma (HCC). A part of the scientific community considers this issue as merely a reflection of a biased interpretation of the data.

Worldwide, HCC accounts for 700,000 new cases every year with a mortality rate of >90%. There are many risk factors associated with it like autoimmune disease, consumption of aflatoxin-contaminated commodities, chronic viral hepatitis caused by hepatitis B and C viruses (HBV and HCV, respectively), and excessive alcohol intake but the link with DAA is the most shocking [1]. It is more concerning because the achievement of SVR should be associated with a decreased risk of cancer. Some studies have mentioned that HCC-induced DAA can be very infiltrative and shows aggressive behavior [4] even at the initial presentation so here we explore if there is any difference between the initial presentation of patients with HCC presenting after DAA or interferon.

We aim to look at the involvement in detail of portal vein thrombosis (PVT), lesion type, staging, and timing of initial presentation between patients treated with DAA and interferon. Initial studies have analyzed the occurrence and recurrence of HCC even after achieving SVR. However, the impact of anti-HCV (DAA and PI) therapy on tumor pattern did not get any attention. Hence, in this study, we aim to explore the relationship of anti-HCV therapy with the time of the initial presentation of HCC and tumor patterns in Pakistan.

## Materials and methods

We conducted a retrospective case-control study of adult patients with HCV-related HCC who achieved SVR after treatment with either DAA or PI. The main outcome was to study if there was any difference between the pattern of HCC in both groups. Also to analyze the mean age of development of HCC after therapy. This comparative study is based on the data of patients diagnosed with HCC as per AASLD (American Association for the Study of Liver Diseases) criteria [5], between January 2019 and March 2020, at the liver centers of a tertiary care hospital located in Rawalpindi, Pakistan. The HCC patients with a history of achieving SVR after treatment with either PEG or DAA were included. 

Patients having a history of infection with any other types of viruses e.g. HBV, HEV, alcohol consumption, and history of any other cancer were excluded. HCV-related HCC patients who received anti-HCV treatment but failed to achieve SVR were also excluded. On the pre-designed Excel sheet, the information regarding demographics, HCV diagnosis, type of anti-HCV treatment, and whether SVR was achieved were included. This information was collected from the patient's current/previous medical records. Based on patients' medical records, the CTP class and BCLC system stage were noted. The enrolled HCC patients were divided into two groups based on the prior treatment they had for HCV; Group A: DAA and Group B: PI group.

## Results

The total number of patients in the DAA group was 37 and the patients in the PI group were 21. The total number of male patients in the DAA group was 62% while either gender was almost equal in PI. The age group of 40-60 was more prevalent in the DAA group while the PI group comprised more patients who were above 60 (Table 1).

**Table 1:** Demographic table. DAA, direct-acting antiviral agent; PI, pegylated-interferon

	DAA	PI
Number of the patient in each group	37	21
Age		
0-40	1 (3%)	0
40-60	24 (65%)	10 (48%)
>60	12 (32%)	11 (52%)
Gender		
Male	23 (62%)	11 (50%)
Female	13 (38%)	10 (50%)

Patients in the DAA group presented after 3.35 years on average while patients in the PI group presented after about seven years. 

Most of the patients presented with the CTP stage A. Per BCLC staging, most of the patients had stage C, which means multiple lesions. Data on vascular involvement were limited so only limited data were included. At the initial presentation, most of the patients presented with multiple lesions.

## Discussion

Our study focuses on the pattern of HCC, timing, and the initial stage at the time of presentation between the DAA and PI group. Our study has revealed that the average time to the presentation of patients after DAA was about 3.35 years, and patients who had undergone the PI course presented after about seven years. According to our results, there is no significant difference between the pattern of spread, number of lesions, and stage at initial presentation between both groups.

Timing

A number of studies have discussed the average time after which HCC initially presents in patients with DAA treatment for HCV. In our study, HCC cases presented to us had DAA therapy on average 3.35 years before (Table 2). On the other hand, patients who had PI for their treatment presented after a mean of seven years as shown in Table 2. HCC was detected in a cohort for up to four years after starting DAA treatment [4]. Another study reported that cases do not show a downtrend of HCC for the first 3.6 years. On the contrary, a study of 139 patients with HCV-related cirrhosis demonstrated a sharp decline in HCC in the second year of follow-up [6].

**Table 2:** Initial presentation with HCC after treatment with either DAA or PEG-interferon. HCC, hepatocellular carcinoma; DAA, direct-acting antiviral agent; PEG, pegylated

Treatment how many years ago	HCC after DAA N(%)	HCC after PEG interferon N(%)
1	3 (8)	1 (5)
2	10 (27)	1 (5)
3	7 (19)	
4	8 (21)	2 (9)
5	8 (22)	2 (9)
6		
7		2 (9)
8	1 (3)	5 (24)
10		5 (24)
11		1 (5)
12		1 (5)

HCC tumor characteristics after DAA treatment

Barcelona Clinic Liver Cancer System classifies patients with HCC into five categories that include: very early, early, intermediate, advanced, and terminal. It incorporates not only data on tumor status, and liver function but also on overall health status. In our case, most of the patients presented had the BCLC stage of C as demonstrated in Table 3. That means that most of the patients at the time of the initial presentation had an intermediate spread of HCC [7]. On the other hand, CTP includes biosynthetic parameters such as circulating albumin, bilirubin, and coagulation characteristics, and subjective parameters such as the presence and severity of ascites and encephalopathy. Most of the patients in our study had a CTP score of A as demonstrated in Table 3. This demonstrates that most of the patients had the least severe liver disease and most of their liver functions were normal [8].

**Table 3:** Demonstrates number and percentage of patients in the staging of CTP and BCLC in each group. Also shows the number of lesions and vascular involvement in both groups. CTP, Child-Turcotte-Pugh; BCLC, Barcelona Clinic Liver Cancer; DAA, direct-acting antiviral agent; PI, pegylated-interferon

	DAA N(%)	PI N(%)
CTP		
A	21 (65)	9 (52)
B	8 (25)	5 (30)
C	3 (9)	3 (17)
BCLC		
A	9 (28)	3 (14)
B	7 (21)	3 (14)
C	14 (43)	8 (38)
D	2 (6)	3 (14)
Vascular involvement		
No	18 (72)	10 (71)
Yes	7 (28)	4 (29)
Number of lesions		
Unifocal	9 (31)	4 (25)
Multifocal	20 (69)	12 (75)

Our study has demonstrated that at first presentation, there was no significant difference between HCC patterns in patients with DAA or interferon therapy. On the other hand, few studies have demonstrated a strong predisposition to aggressive disease in the case of prior DAA use. A study demonstrated that the number of infiltrative PVT, HCC pattern, and regional lymph node metastasis was significantly higher among HCC patients after DAA treatment than among those without DAA (p ≤ 0.05). HCC patients after DAA treatment were found to have a significantly higher malignant portal vein invasion and local spread through malignant lymphadenopathy [9]. Another prospective study showed similar findings of significant multifocal and infiltrative HCC patterns after DAA treatment [4]. Romano et al. suggested that tumor growth in patients with aggressive tumors after HCV therapy is faster and with a higher number of nodules and extra-hepatic metastases [10].

Limitations of our study include the retrospective design and a small data set of 58 patients. The strengths of this study include a case-control approach in understanding HCC patterns in HCV patients with prior treatment with either PI or DAA.

## Conclusions

In conclusion, the pattern of HCC did not differ between patients treated with PI and those treated with DAAs. The issue of HCC incidence and progression after the cure of hepatitis C is unresolved. The most important thing to keep in our mind is that chronic hepatitis C infection predisposes the patient to HCC and liver damage persists and progresses despite treatment rather than being brought on by the medications. Further studies need to be done to fully understand if it is a mere coincidence or a result of lifesaving yet dangerous medicine.
